# *Mycobacterium tuberculosis* replicates within necrotic human macrophages

**DOI:** 10.1083/jcb.201603040

**Published:** 2017-03-06

**Authors:** Thomas R. Lerner, Sophie Borel, Daniel J. Greenwood, Urska Repnik, Matthew R.G. Russell, Susanne Herbst, Martin L. Jones, Lucy M. Collinson, Gareth Griffiths, Maximiliano G. Gutierrez

**Affiliations:** 1 Host-Pathogen Interactions in Tuberculosis Laboratory, The Francis Crick Institute, London NW1 1AT, England, UK; 2 Electron Microscopy Science Technology Platform, The Francis Crick Institute, London NW1 1AT, England, UK; 3 Department of Biosciences, University of Oslo, 0371 Oslo, Norway

## Abstract

*Mycobacterium tuberculosis* modulation of macrophage cell death is a well-documented phenomenon, but its role during bacterial replication is less characterized. In this study, we investigate the impact of plasma membrane (PM) integrity on bacterial replication in different functional populations of human primary macrophages. We discovered that IFN-γ enhanced bacterial replication in macrophage colony-stimulating factor–differentiated macrophages more than in granulocyte–macrophage colony-stimulating factor–differentiated macrophages. We show that permissiveness in the different populations of macrophages to bacterial growth is the result of a differential ability to preserve PM integrity. By combining live-cell imaging, correlative light electron microscopy, and single-cell analysis, we found that after infection, a population of macrophages became necrotic, providing a niche for *M. tuberculosis* replication before escaping into the extracellular milieu. Thus, in addition to bacterial dissemination, necrotic cells provide first a niche for bacterial replication. Our results are relevant to understanding the environment of *M. tuberculosis* replication in the host.

## Abstract (precis)

*Mycobacterium tuberculosis* triggers macrophage cell death by necrosis, but it is unclear how this affects bacterial replication. Lerner et al. show that this pathogen replicates within necrotic human macrophages before disseminating to other cells upon loss of plasma membrane integrity.

## Introduction

The intracellular lifestyle of *Mycobacterium tuberculosis* represents a crucial stage in the pathogenesis of tuberculosis, and successful drug discovery programs have to include in vitro studies using infected host cells (Young et al., 2008; Lechartier et al., 2014). Most of the in vitro studies of infection with *M. tuberculosis* rely on infected macrophages and survival analysis by colony-forming units (CFUs), luciferase, or fluorescent reporters. The use of primary human macrophages is of clear advantage because there are some fundamental differences between human macrophages and those originating from other species. Important differences are obvious, such as the lack of a large set of IFN-γ–inducible GTPases that are exclusively present in mouse macrophages (Kim et al., 2012) or the differential production of nitric oxide when compared with mouse macrophages (Thomas and Mattila, 2014).

In vivo, macrophages are composed of a very heterogeneous population as a result of multiple differentiation and activation stimuli present in tissues (Martinez and Gordon, 2014). Numerous experimental approaches are used to differentiate human macrophages in vitro with the aim of mimicking the heterogeneity present in tissue macrophages during immunity and inflammation (Vogt and Nathan, 2011; Martinez and Gordon, 2015). Macrophages differentiated in vitro are often referred to as classical or alternatively activated macrophages, or M1 and M2 macrophages, respectively. Several stimuli are used to differentiate macrophages in vitro, and in this context, granulocyte–macrophage colony-stimulating factor (GM-CSF; considered M1)– and macrophage colony-stimulating factor (M-CSF; considered M2)–differentiated human macrophages are widely used models of macrophage biology (Lacey et al., 2012; Martinez and Gordon, 2015). GM-CSF macrophages are generally proinflammatory and display enhanced antigen presentation, phagocytosis, and microbicidal capacity. However, M-CSF macrophages display an antiinflammatory cytokine profile after stimulation (Lacey et al., 2012; Martinez and Gordon, 2014). Although GM-CSF– and M-CSF–differentiated macrophages clearly respond differently to extracellular stimuli (Lacey et al., 2012), the nature of this differential response is less clear.

Adding more complexity to in vitro systems of infection, there are a wide range of methods described in the literature to activate macrophages. One of the key cytokines used for the activation of human macrophages is IFN-γ. This cytokine is a key modulator of the phagocytic and mycobactericidal activity of mouse macrophages (Flynn and Chan, 2001). Although studies show that genetic errors of IFN-γ immunity result in severe tuberculosis in children (Abel et al., 2014), the role of IFN-γ in the antimycobacterial activity of human macrophages and its role in pulmonary tuberculosis in adults is still unclear (Abel et al., 2014; Lerner et al., 2015).

Although in vitro and in vivo studies highlight the importance of host cell death modes during mycobacterial control or dissemination, the mode of host cell death in the pathogenesis of human tuberculosis is not completely understood. In tuberculosis, apoptosis is generally considered to be a part of a host protective response, whereas necrosis is considered to be a pathway for bacterial dissemination and granuloma cavity formation (Behar et al., 2010; Ramakrishnan, 2012; Wong and Jacobs, 2016). Apoptosis is believed to help with the eradication of *M. tuberculosis* (Keane et al., 2000; Behar et al., 2010), and, unsurprisingly, this pathogen has strategies to inhibit apoptosis (Velmurugan et al., 2007). Apoptosis is protective in part because bacteria are internalized via efferocytosis and subsequently eliminated (Martin et al., 2012). However, necrosis of infected cells helps bacterial dissemination. *M. tuberculosis* inhibits the plasma membrane (PM) repair pathway, resulting in the progression to necrosis and mycobacterial release into the extracellular environment (Divangahi et al., 2009). Nevertheless, whether host cell necrosis directly affects bacterial replication has not yet been demonstrated.

In this study, we show that *M. tuberculosis* replicates to a similar extent in GM-CSF– and M-CSF–differentiated macrophages (GM-CSF macrophages and M-CSF macrophages, respectively). Remarkably, IFN-γ activation enhanced *M. tuberculosis* replication in M-CSF macrophages but not in GM-CSF macrophages, and this correlated with an increased susceptibility to necrosis compared with the other macrophage populations. Long-term live-cell imaging of infected macrophages revealed that at the single-cell level, *M. tuberculosis* infection induces loss of PM integrity to replicate in damaged cells before entering the extracellular milieu. Collectively, our data define a differential susceptibility to the necrosis of GM-CSF– and M-CSF–differentiated macrophages after infection and IFN-γ activation. Importantly, these differences are critical for the replication of *M. tuberculosis* in damaged cells before dissemination.

## Results and discussion

### *M. tuberculosis* replicates in both resting and IFN-γ–activated GM-CSF– and M-CSF–differentiated human macrophages

**Figure 1.**
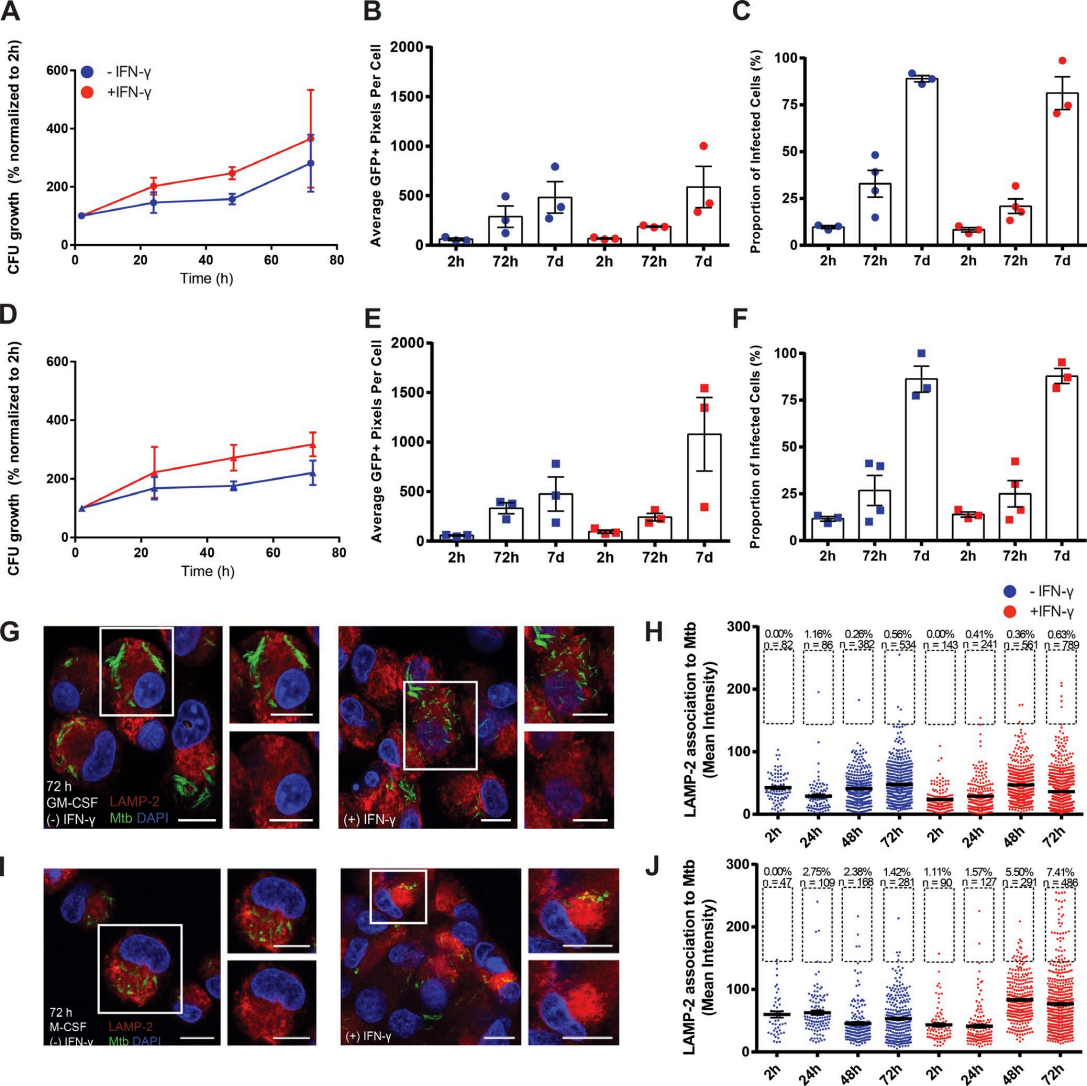
**Dynamic growth of *M. tuberculosis* in distinct subpopulations of human primary macrophages.** (A) Intracellular replication of GFP-Mtb in resting (blue line) or IFN-γ–activated (red line) GM-CSF– differentiated macrophages, normalized to 2 h and expressed as the percent increase in CFUs over time. (B) Intracellular replication of GFP-Mtb in resting (blue dots) or IFN-γ–activated (red dots) GM-CSF–differentiated macrophages (expressed as the mean number of GFP-positive pixels per cell) at 2 h, 72 h, and 7 d after infection. (C) Quantification of the mean proportion of GFP-Mtb infected cells in resting (blue dots) or activated (red dots) GM-CSF–differentiated macrophages at 2 h, 72 h, and 7 d after infection. (D–F) Same as in A–C, but with M-CSF–differentiated macrophages. (A–F) Data presented are means ± SEM from three (A, B, D, and E) or at least three (C and F) independent experiments. (G) Analysis of the association of GFP-Mtb with LAMP-2 in resting and activated GM-CSF macrophages infected at an MOI of 1 for 72 h. Representative confocal microscopy images of GFP-Mtb and LAMP-2 marker. Nuclei were stained with DAPI. Insets show magnifications of the outlined cells, highlighting the low level of association of LAMP-2 to bacteria. (H) Quantification of the association of GFP-Mtb with LAMP-2 in resting (blue) or activated (red) GM-CSF macrophages. The number of bacteria analyzed for each condition is displayed as well as the proportions of *M. tuberculosis* that are considered to be positive for LAMP-2 association (i.e., a mean intensity value of ≥150). (I and J) Same as G and H, but with M-CSF–differentiated macrophages. Bars, 10 µm.

First, we characterized each macrophage subset by flow cytometry using surface markers of activation. Then, we verified that they showed a mature phenotype (CD86 positive; Fig. S1 A) and were responsive to IFN-γ by looking at the expression of the IFN-γ receptor (Fig. S1 B) and by inducing indoleamine 2,3-dioxygenase (IDO) and other IFN-γ–inducible proteins (Fig. S1 C and not depicted). Next, we analyzed the replication of *M. tuberculosis* H37Rv expressing EGFP (GFP-Mtb) in GM-CSF human primary macrophages by CFUs. We observed that on average, bacteria replicated by ∼2.5-fold after 72 h of infection (Fig. 1 A). Beyond the 72-h time point, it was not possible to determine intracellular bacterial replication using CFUs because the dying cells detached, resulting in an underestimation of the actual number of intracellular bacteria in the sample. Therefore, to analyze longer periods of infection, we used a microscopy-based method, which more closely reflected how the infection progressed over an extended in vitro infection at the single-cell level. A quantitative analysis of the number of GFP-positive pixels per macrophage confirmed that *M. tuberculosis* replicated over time at the single-cell level (Fig. 1 B). In parallel, the proportion of *M. tuberculosis–*infected cells per sample significantly increased between hour 2 and day 7, suggesting that the infection was spreading between cells (Fig. 1 C). Activation of GM-CSF macrophages with IFN-γ had only a limited effect on bacterial replication (Fig. 1, A–C). We observed a similar level of *M. tuberculosis* replication in M-CSF compared with GM-CSF macrophages by CFUs (Fig. 1 D) and also by microscopy at the single-cell level (Fig. 1, E and F). Interestingly, we observed that at 7 d after infection in IFN-γ–activated M-CSF macrophages, the mean burden of *M. tuberculosis* per cell was much higher than all of the other conditions (Fig. 1, B and E). Furthermore, infection of macrophages with GFP-Mtb ΔRD1 (which lacks the ESX-1 type VII secretion system; Fig. S2, A–D) revealed that this mutant strain had a reduced bacterial burden (Fig. S2, A and D) and a reduced cell-to-cell spread (Fig. S2 B) compared with wild type and lacked the ability to induce cell death overall (Fig. S2 C), even after 5 d of infection (Fig. S2 D and Video 1). Bacterial viability was independent of the time of activation or IFN-γ concentration because prestimulation overnight or treatment after infection with a higher concentration of IFN-γ rendered similar results (not depicted). In resting GM-CSF macrophages, bacteria were mostly localized in compartments negative for the phagolysosomal marker LAMP-2, as was expected for bacteria residing in an arrested early phagosome (Fig. 1, G and H). IFN-γ activation did not significantly change this localization (Fig. 1, G and H). In M-CSF macrophages, *M. tuberculosis* was also localized in LAMP-2–negative compartments (Fig. 1, I and J), but IFN-γ increased the fraction of mycobacteria found in late endocytic compartments after 48 h of infection (Fig. 1, I and J). Overall, our results are in agreement with a study indicating that in GM-CSF and M-CSF macrophages, *M. tuberculosis* grows at similar rates (Tailleux et al., 2003), but our results are also in disagreement with other studies (Douvas et al., 1985; Akagawa et al., 2006; Wong and Jacobs, 2013). It is likely that differences in donor samples, mycobacterial strains, MOI, and also cell differentiation protocols are explanations for discrepancies among studies. However, our data highlight important physiological differences between GM-CSF and M-CSF macrophages during the late stages of *M. tuberculosis* infection in vitro. We suggest that the alveolar macrophage phenotype conferred by GM-CSF (Akagawa, 2002) makes the macrophages permissive to *M. tuberculosis* replication, allowing bacterial dissemination after primary infection of the lungs. In contrast, in M-CSF macrophages, IFN-γ will promote bacterial replication and, later on, dissemination. The interplay of these differentiation factors, their contribution to bacterial replication and dissemination in vivo, and the physiological differences between human and mouse macrophages remain to be investigated. Although we observed variability across the donors tested in this work, IFN-γ unexpectedly did not have any major impact on bacterial growth in GM-CSF macrophages in vitro. It is not clear why IFN-γ did not control bacterial replication in our system compared with other studies. A possible cause is that our study was not performed under hypoxic conditions, which were shown to be antimycobacterial (Vogt and Nathan, 2011). Also, reported differences in nitric oxide production between human and mouse macrophages can account for reduced potency in human cells (Thomas and Mattila, 2014). Our data are consistent with a study showing that in human macrophages at 5–10% O_2_, IFN-γ induced the growth of mycobacteria and the induction of macrophage extracellular traps (Wong and Jacobs, 2013). Our data are also consistent with early studies showing that IFN-γ enhanced *M. tuberculosis* growth (Douvas et al., 1985), although we were not able to detect macrophage extracellular traps in our system. Our data are also in agreement with studies in mouse macrophages showing that when a threshold of intracellular bacteria is reached, IFN-γ had little influence on bacterial growth (Repasy et al., 2013). Additionally, studies in human patients—particularly children—have shown that IFN-γ is a key antimycobacterial cytokine (Abel et al., 2014). It remains to be defined which in vitro systems with human macrophages better capitulate macrophage biology in vivo. In this context, cytokines such as IFN-γ could be affecting other cell types with potential relevance to the pathology of human tuberculosis (Lerner et al., 2015).

### *M. tuberculosis* replication continues after the loss of PM permeability

**Figure 2.**
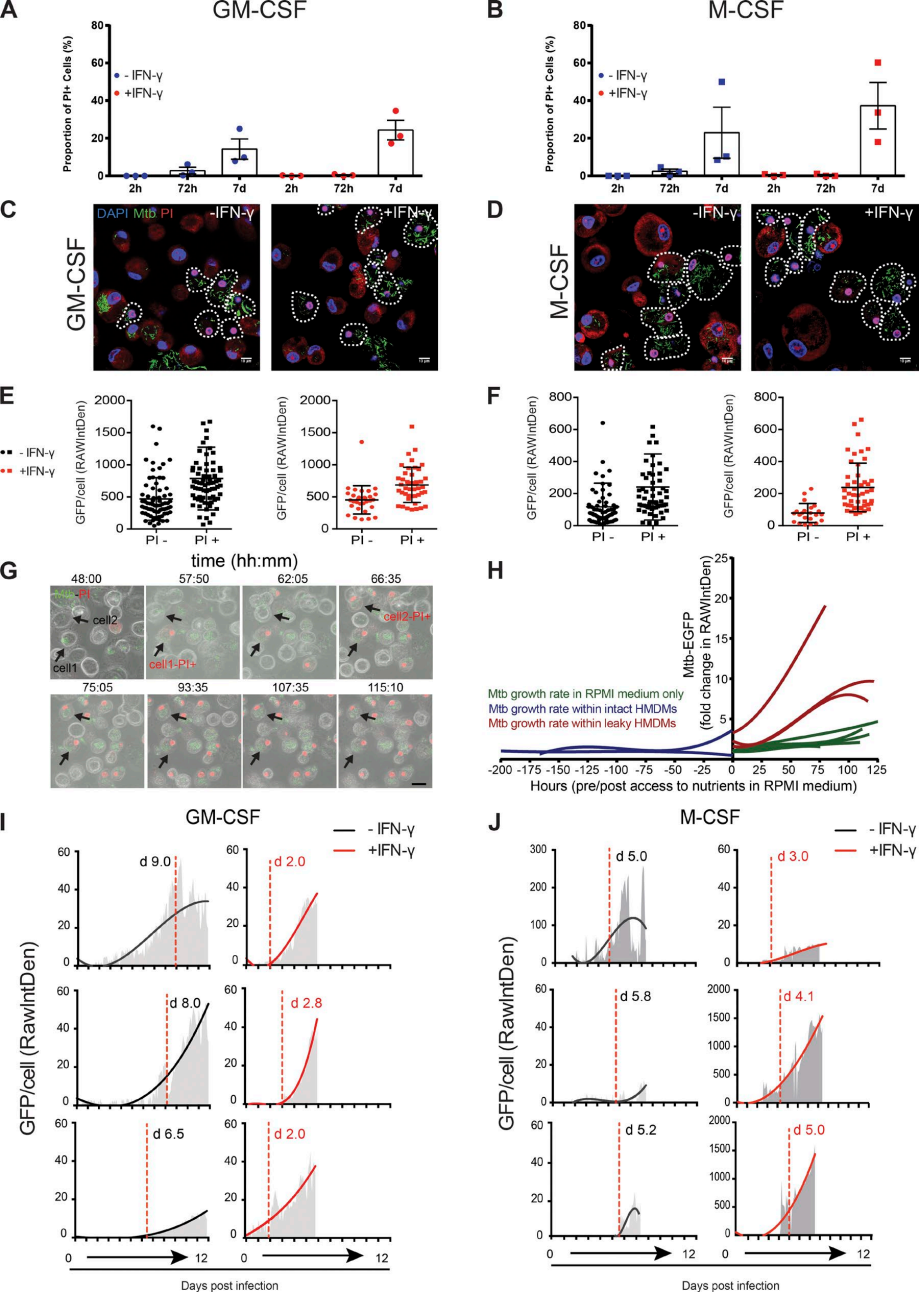
**Differential susceptibility to necrosis in macrophages leads to *M. tuberculosis* growth.** (A) Quantification of the proportion of cells that had a PI-positive (PI+) nucleus in resting (blue dots) or activated (red dots) GM-CSF macrophages. A PI-positive nucleus indicates that the cell membrane has become permeable to the extracellular medium and therefore is likely to be dead. Each data point represents one independent experiment, and the bars show means ± SEM. (B) Same as in A, but with M-CSF macrophages. (C) Representative confocal images of macrophages infected with GFP-Mtb (green) and labeled with PI (red). Macrophages were infected at an MOI of 1 for 2 h. 7 d after infection, macrophages were stained with 25 µg/ml PI in RPMI for 15 min and fixed. PI-positive cells are outlined. (D) Same as in C, but with M-CSF macrophages. Bars, 10 µm. (E) Quantification of GFP-Mtb replication in PI-negative or PI-positive cells in resting (black squares) or activated (red squares) GM-CSF macrophages. Images were analyzed, and the GFP signal per cell was plotted. Data represent the means ± SEM of one representative experiment. (F) Same as in E, but with M-CSF macrophages. (G) Representative snapshots at the indicated time points (Video 2) of resting GM-CSF macrophages infected with GFP-Mtb (green) in the presence of PI. Macrophages were infected at an MOI of 1 for 2 h, and after 24-h infection, the media were replaced with RPMI medium containing 0.4 µg/ml PI. Cells were imaged for at least 7 d. Bar, 20 µm. (H) Comparison of GFP-Mtb growth rates as measured by live-cell imaging. Three conditions were tested: *M. tuberculosis* in RPMI medium alone (green lines), *M. tuberculosis* within GM-CSF–differentiated human monocyte-derived macrophages (HMDMs) before the moment the cell becomes PI positive (blue lines), and *M. tuberculosis* after human monocyte-derived macrophages become PI positive (red lines). Each line shows the growth rate of *M. tuberculosis* represented by the fold change in GFP signal (RAWIntDen) of the GFP-Mtb signal over time, normalized to the first frame in the video. (I) Quantification of intracellular replication of GFP-Mtb before and after membrane damage in resting or activated GM-CSF and M-CSF macrophages at the single-cell level. Red dashed lines indicate when the cell’s nucleus becomes PI positive. GFP-Mtb signal was monitored in three representative cells from each condition using ImageJ software. (J) Same as in I, but with M-CSF macrophages.

We then focused on understanding the differential effect of IFN-γ in enhancing the replication of *M. tuberculosis* in M-CSF macrophages compared with GM-CSF macrophages. To monitor the proportion of cells that had lost PM integrity, we stained the cells with propidium iodide (PI) before fixation (Fig. 2, A and B). We observed relatively low levels of cell death in any condition up to 72 h after infection, but by day 7 after infection, cell death was apparent to differing extents in the macrophage populations (Fig. 2, A and B). Although there was noticeable variation between donors, activated M-CSF macrophages had the largest mean proportion of PI-positive cells, with approximately double the mean of the other macrophage populations (Fig. 2, A and B). We observed that in PI-positive cells at 7 d after infection, the bacterial burden was higher in both GM-CSF (Fig. 2, C and E) and M-CSF (Fig. 2, D and F) macrophages in both resting and activated conditions. We therefore hypothesized that these necrotic cells were providing a niche for *M. tuberculosis* replication. Using microscopy with fixed samples provided only snapshots in time, which meant that we could not determine whether the cells were dying because of the high bacterial burden or if the cells became more permissive for replication after they became necrotic. To investigate these dynamic events in more detail, we performed live-cell imaging of infected cells with the presence of PI in the medium to monitor macrophage PM integrity. We observed that when macrophages became leaky (positive for PI), bacterial replication continued in necrotic cells that nevertheless retained their cellular morphology (Fig. 2 G and Video 2). Continued *M. tuberculosis* replication after a loss of PM integrity is not solely caused by *M. tuberculosis* gaining access to and obtaining nutrients from the extracellular RPMI medium because the growth rate in leaky macrophages (Fig. 2 H, red lines) was increased compared with the growth rate in medium alone (Fig. 2 H, green lines). A quantitative analysis of GFP fluorescence intensity at the single-cell level suggests that in a proportion of macrophages, detectable mycobacterial replication does not begin until after the cells have become leaky (Fig. 2, I and J). Bacterial replication was observed in both resting and activated GM-CSF macrophages (Fig. 2 I), consistent with the previous data (Fig. 1, A–F). In resting M-CSF macrophages, mycobacterial replication started after macrophages became leaky (Fig. 2 J). However, IFN-γ activation accelerated both PM damage and bacterial cell growth (Fig. 2, I and J). We concluded that during in vitro infection, loss of PM integrity permits continued *M. tuberculosis* replication and that the extent of cell death in the sample correlates with the total bacterial burden. Thus, IFN-γ enhanced PM damage (without causing significant chromatin decondensation) in M-CSF macrophages, which allowed greater bacterial replication per macrophage. This is consistent with data showing that fast replicating bacteria induced more necrosis in vivo (Repasy et al., 2013, 2015). Alternatively, IFN-γ could regulate macrophage cell death and thereby bacterial replication, as shown for mouse macrophages (Lee and Kornfeld, 2010).

### Necrotic macrophages provide a niche for *M. tuberculosis* replication

**Figure 3.**
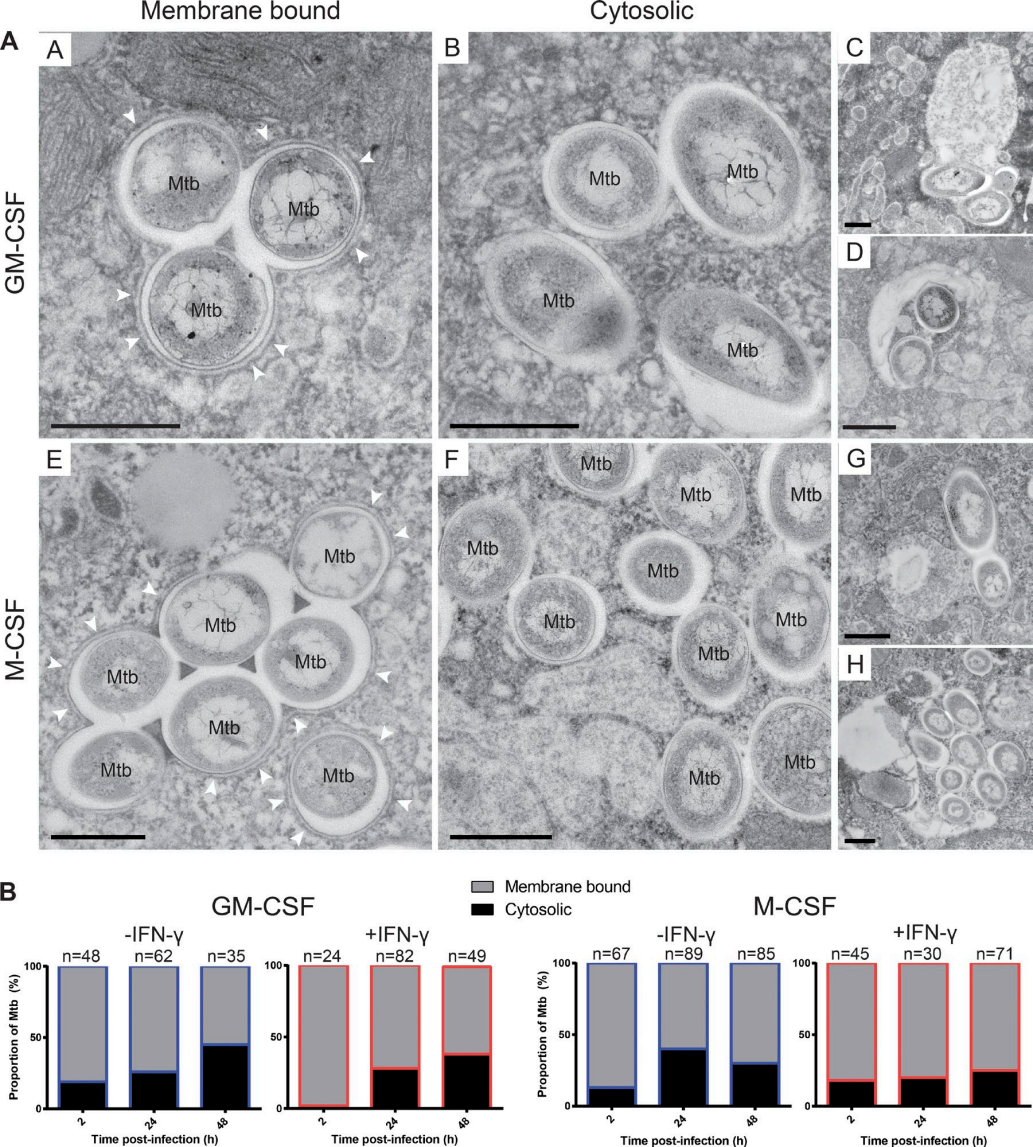
***M. tuberculosis* is localized in the cytosol of primary human macrophages.** (A) TEM images representing the observed localization of *M. tuberculosis* in GM-CSF– and M-CSF–differentiated macrophages at 48 h after infection. “Membrane bound” represents bacteria in a phagosome (with one surrounding host membrane, such as in subpanels A and E, highlighted by white arrowheads), bacteria in phagolysosomes (loose vesicles with one surrounding host membrane, such as in subpanels C and G), or bacteria in autophagosomes (with two or more surrounding host membranes, such as in subpanels D and H); “Cytosol” represents bacteria with no surrounding host membranes (such as in subpanels B and F). Bars, 500 nm. (B) Quantification of GFP-Mtb subcellular localizations in resting (blue-outlined bars) or activated (red-outlined bars) GM-CSF and M-CSF macrophages at 2, 24, and 48 h after infection. Quantification was performed by stereological analysis of TEM images of resin sections of infected macrophages. Data are from one experiment, and the total numbers of cells analyzed in each condition (*n*) are shown.

**Figure 4.**
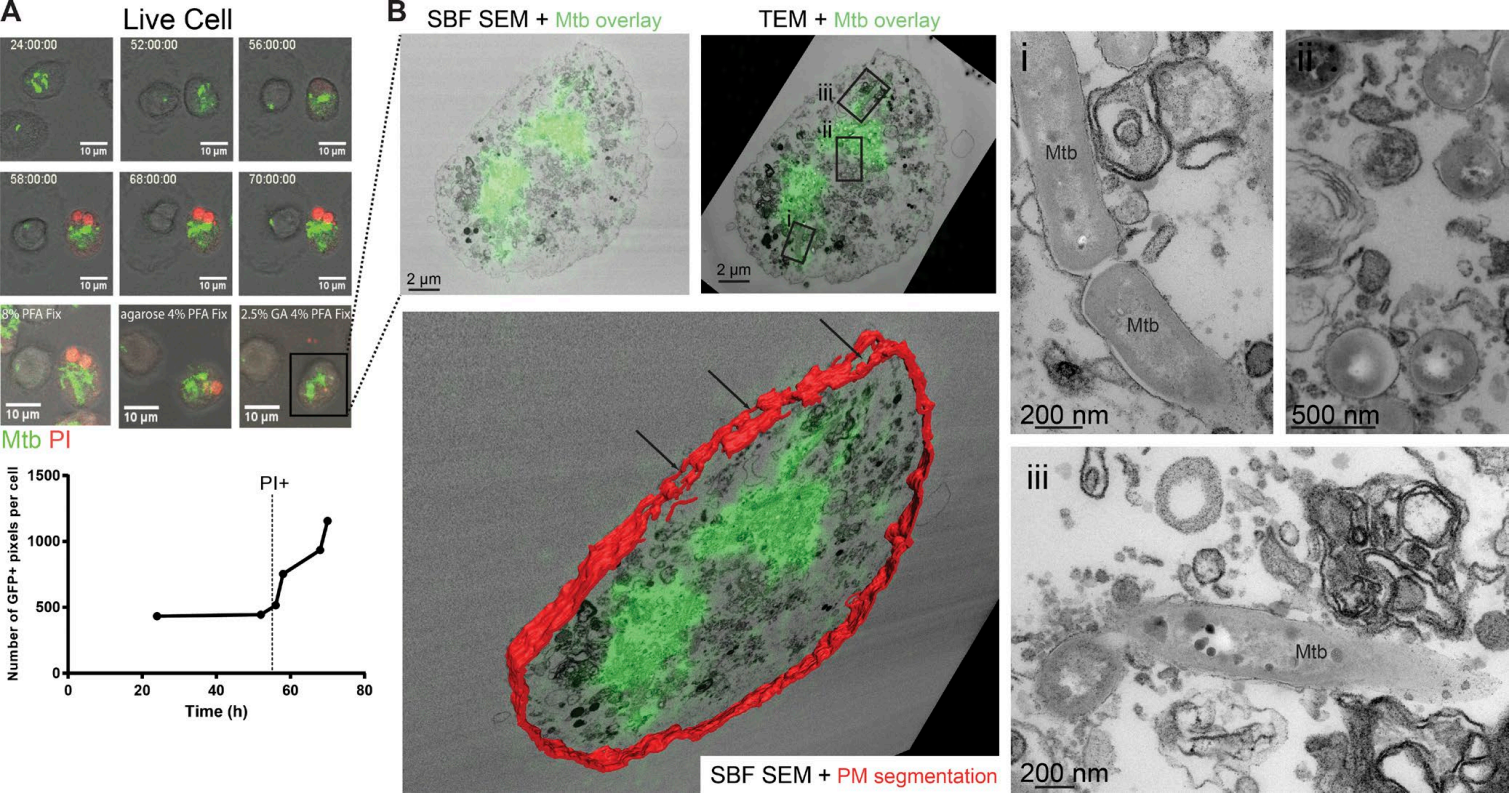
***M. tuberculosis* replicates in a cellular necrotic environment.** (A) Snapshots taken from live-cell imaging of GM-CSF macrophages infected with GFP-Mtb over 4 d; the graph below shows how the growth of GFP-Mtb occurs only after the cell becomes PI positive. (B) SBF SEM and TEM were performed on the same sample as in A. This allowed for the GFP-Mtb signal to be overlaid with the EM images. Subpanels i–iii show magnified regions of the ultrastructure taken from the TEM image. The bottom subpanel shows segmentation of the PM in red using the SBF SEM data (with reference to the adjacent TEM image) with the LM data and TEM image overlay. Black arrows indicate PM damage.

We next focused on characterizing the environment where *M. tuberculosis* replicates in human macrophages. Because *M. tuberculosis* can be localized in the cytosol under some conditions (van der Wel et al., 2007), we performed an ultrastructural analysis of *M. tuberculosis* localization using EM during the growth of *M. tuberculosis* in GM-CSF and M-CSF macrophages, as described previously (Fig. 3; Lerner et al., 2016). Resting GM-CSF macrophages had an increased proportion of bacteria present in the cytosol compared with M-CSF after 48 h (45% and 30%, respectively; Fig. 3 B), and IFN-γ activation did not affect the ultimate localization of the bacteria (Fig. 3 B). It was interesting to note that after 5 h plus 2 h of infection, there was already up to 15% of the bacteria present in the cytosol in both GM-CSF and M-CSF macrophages (Fig. 3 A, subpanels B and F; and Fig. S2 E). To further characterize at the ultrastructural level the environment where *M. tuberculosis* replicated, we followed *M. tuberculosis* growth by live-cell imaging as described previously and performed correlative live-cell and electron microscopy (CLEM; Fig. 4; Russell et al., 2016). As shown by live-cell imaging, after 56 h of infection, the PM became permeable and bacteria started to replicate (Fig. 4 A). At 70 h after infection, cells were fixed and processed for EM. Although the multistage fixation protocol affected the morphology of the cell, we imaged after each step to document the changes (Fig. 4 A). By morphology, we could confirm that *M. tuberculosis* was growing in a necrotic cell with features of late necrotic cell death that include PM damage, swollen and dilated organelles, as well as autophagic structures (Fig. 4 B). We observed that replicating bacteria were in close apposition to host membranes and were exposed to an environment rich in cellular membranes and undigested organelles. Collectively, already at 48 h, a large proportion of the bacilli were found in the cytosol, and then at later stages, mycobacteria were mostly replicating in an early to late necrotic macrophage environment.

### Inhibition of host cell necrosis limits *M. tuberculosis* replication

**Figure 5.**
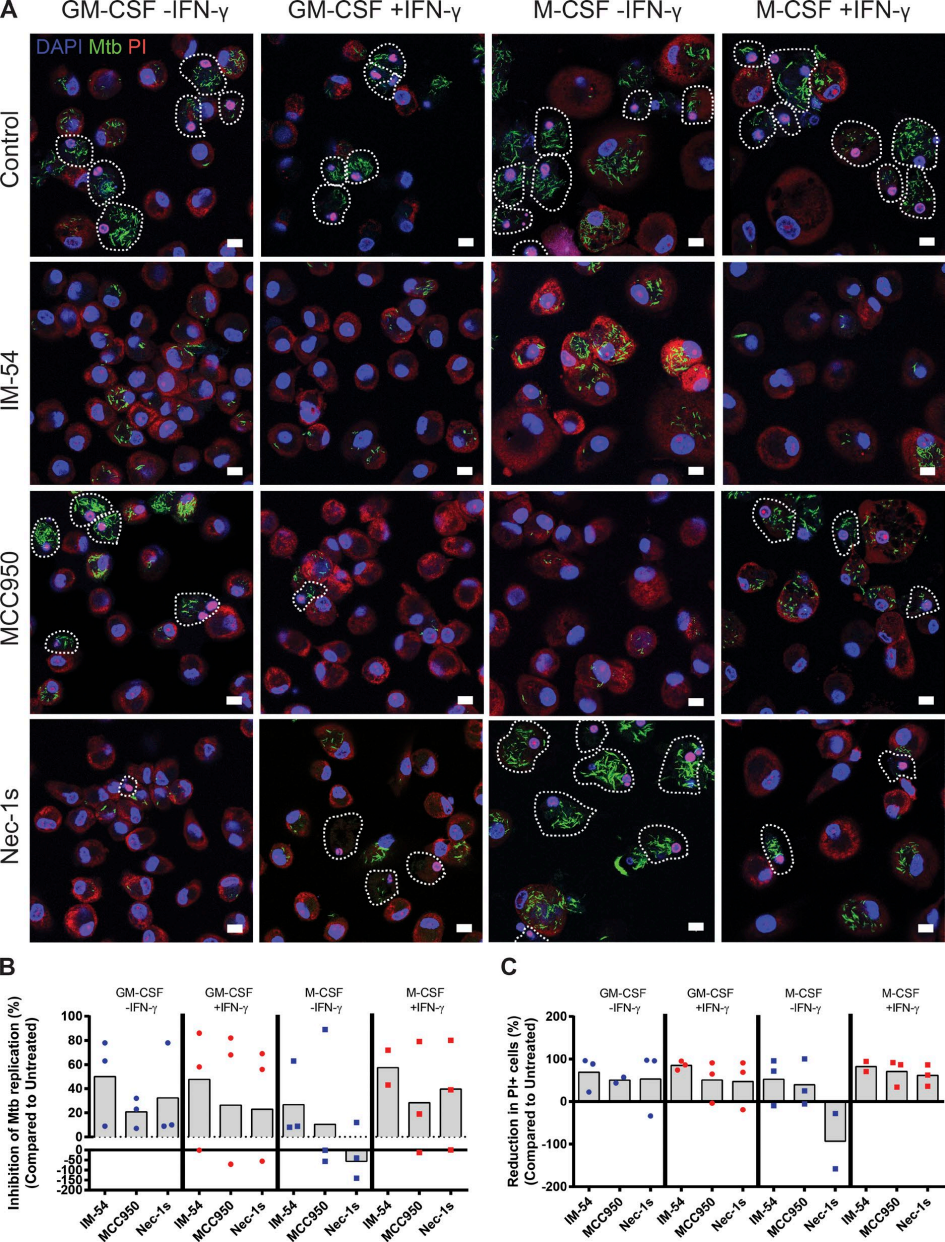
**Inhibition of host cell necrosis limits *M. tuberculosis* replication.** (A) Example confocal microscopy images showing GFP-Mtb (green), PI (red), and DAPI (blue) staining of resting or activated GM-CSF or M-CSF macrophages at 7 d after infection in control cells (DMSO only) or cells treated with 10 µM of the shown necrosis inhibitors. Cells outlined in white dotted lines are considered to be PI positive. Bars, 10 µm. (B) Quantification of the effect of treating infected resting (blue) or activated (red) GM-CSF (dots) or M-CSF (squares) with three necrosis inhibitors compared with untreated control macrophages at 7 d after infection with GFP-Mtb. (C) Quantification of the effect of treating infected resting (blue) or activated (red) GM-CSF (dots) or M-CSF (squares) with three necrosis inhibitors on the proportion of cells with a PI-positive (PI+) nucleus compared with untreated control macrophages at 7 d after infection with GFP-Mtb. Data points show the percent reduction for each independent experiment, and the bars show the overall mean.

Because *M. tuberculosis* most efficiently grows within necrotic cells, we reasoned that we should be able to modulate bacterial replication using chemical inhibitors of necrosis. We used three inhibitors: IM-54, a potent inhibitor of oxidative stress–induced necrosis (Dodo et al., 2005); MCC950, a specific inhibitor of the NLRP3 inflammasome (Coll et al., 2015); and Necrostatin-1s (Nec-1s), a RIP1 kinase (RIPK1) inhibitor (Degterev et al., 2013). Resting and activated GM-CSF and M-CSF macrophages were infected with GFP-Mtb for 7 d (Fig. 5 A), and the percent inhibition of *M. tuberculosis* replication was determined (Fig. 5 B). In parallel, the proportion of PI-positive cells was measured to confirm that the inhibitors were indeed preventing cell death (Fig. 5 C). We also confirmed that the inhibitors themselves did not affect *M. tuberculosis* growth at the same concentration in vitro (Fig. S3 A). Notably, all of the drug treatments inhibited to differing extents the mean *M. tuberculosis* replication compared with the DMSO control in each macrophage population analyzed (Fig. 5, A and B). Interestingly, Nec-1s treatment in resting M-CSF macrophages increased the mean bacterial burden; consistent with this, in this particular condition, Nec-1s actually increased the proportion of PI-positive cells (Fig. 5 C). This suggests that RIPK1 could be differentially activated in the different macrophage populations. The lack of effect on cell death and bacterial replication in M-CSF macrophages was reversed by IFN-γ activation, indicating that this cytokine activates in macrophages a signaling pathway via RIPK1 that results in necrosis, as was previously reported in fibroblasts (Thapa et al., 2013). The precise mechanism and signaling pathways that regulate this differential susceptibility to PM integrity after infection and IFN-γ activation between M-CSF and GM-CSF macrophages remains to be investigated. Importantly, the necrosis inhibitors were more efficient in activated than in resting M-CSF macrophages, correlating with the increased replication and enhanced cell death observed (Figs. 1 E and 2 B). Thus, we concluded that inhibition of necrosis resulted in less efficient *M. tuberculosis* growth.

Our results highlight the possibility that bacteria replicate within cells after PM damage before being released into the extracellular medium. If this scenario holds in vivo, extracellular bacteria would originate from two different sources: the pool that replicated intracellularly in damaged cells and a pool of bacteria that grows extracellularly. Necrotic cells represent a much better environment for bacterial replication, as they are rich in nutrients in a host cell with severely affected cell-autonomous immunity. Our data are consistent with the idea that virulent mycobacteria inhibits the cell PM repair program, thereby inducing necrosis and a release of bacteria that can then reinfect surrounding cells (Divangahi et al., 2009). In addition, our data revealed that *M. tuberculosis* induces PM damage, seemingly to disarm the host cell, and then replicates in this nutrient-rich environment. Once replication occurs in this protected and rich environment, bacteria immediately get access to the extracellular media after reinfection. Our data therefore provide evidence suggesting that *M. tuberculosis* actively avoid the extracellular environment that can be detrimental for long periods of time. A study performed with a high burden of bacterial infection (Lee et al., 2006) could mimic some of our results, but our experimental setting is different and could reflect more the cells that in vivo are infected with fewer bacteria and not the ones that take up large bacterial aggregates. In our system, we observed by live-cell imaging that once macrophages internalized big clumps of bacteria (either by cell transfer or efferocytosis), cell death followed rapidly (unpublished data). Collectively, in this study, we analyzed for the first time the dynamic replication of *M. tuberculosis* in human macrophages by long-term live-cell imaging in different functional populations of macrophages. Our studies provide insights into the dynamic interactions between human macrophages and *M. tuberculosis*. Thus, in addition to the known role of dissemination, leaky cells undergoing early stages of necrosis represent a niche that helps bacteria to grow even before getting freely into the extracellular environment. Our studies uncover an important aspect in the biology of tuberculosis, revealing that *M. tuberculosis* continues to replicate in host cells after PM integrity is lost before disseminating to other cells.

## Materials and methods

### Preparation of monocyte-derived macrophages

Peripheral blood mononuclear cells (PBMCs) from healthy anonymous donors were isolated from leukocyte provided by the National Blood and Transplant Service, UK. Red blood cells were removed by centrifugation on Ficoll-Paque (28-4039-56 AD; GE Healthcare) and red cell lysis buffer (11814389001; Sigma-Aldrich). Recovered PBMCs were washed to remove platelets. Monocytes were isolated from PBMCs using a magnetic cell separation system with anti-CD14 mAb-coated microbeads (130-050-201; Miltenyi Biotec). CD14-positive monocytes were cultured in complete RPMI 1640 medium (Lonza) supplemented with 9.1% heat-inactivated FCS supplemented with either 50 ng/ml GM-CSF or 50 ng/ml M-CSF (130-093-862/130-093-963; Miltenyi Biotec) at 37°C under a humidified 5% CO_2_ atmosphere for 6 d. Media were replaced at 3 d. On day 6, the cells were washed and detached with 0.5 mM EDTA in ice-cold PBS and plated in a 24-well plate (containing coverslips for microscopy) or a live-cell imaging dish at a concentration of 2 × 10^5^ cells/well with complete RPMI media (for resting macrophages). Macrophages were either treated overnight or after infection with recombinant human IFN-γ (PHC 4031; Gibco) at a concentration of 100 U/ml.

### Bacterial culture

GFP-Mtb or *M. tuberculosis* H37Rv ΔRD1 expressing EGFP (GFP-Mtb ΔRD1) was grown at 37°C in Middlebrook 7H9 medium (Difco Laboratories) containing 0.5% glycerol and 0.05% Tween 80 and was supplemented with 10% oleic acid–albumin–dextrose–catalase supplement (OADC; BD) or on Middlebrook 7H10 plates (Difco Laboratories) supplemented with 10% OADC.

### Infection of human monocyte-derived macrophages

Macrophages were seeded onto 24-well culture plates the day before the infection. *M. tuberculosis* cultures were grown to the midexponential phase. On the day of infection, the bacteria were pelleted and washed with PBS and RPMI/10% FCS at 3,000 rpm for 5 min. An equal volume of sterile glass beads (2.5–3.5 mm) that matched the pellet size was added (usually three to four beads) and then vigorously shaken for 1 min to break up bacterial clumps. The bacteria were washed again with complete RPMI at 1,200 rpm for 5 min. The supernatant was transferred into a new tube, and the measured OD_600_ was then diluted to 0.1 in complete RPMI. Next, cells were infected with M. tuberculosis at an MOI of 1 for 2 h and then washed once with PBS and incubated with complete RPMI media containing IFN-γ and/or 10 µM of the necrosis inhibitors IM-54 (Sigma-Aldrich), MCC950 (Cayman Chemicals), or Nec-1s (Source Biosciences), where relevant.

### CFU determination

For counting bacterial viability, macrophages were washed once with PBS and lysed with water–Tween 80 (0.05%) for 30 min at room temperature. The lysed solution from triplicate wells was taken and serially diluted in PBS in 10-fold steps until 1:1,000 dilution. 25 µl from each dilution was plated in triplicate onto complete 7H11 agar plates. Agar plates were incubated for 2–3 wk at 37°C. Triplicate plates were averaged, and CFU were calculated and plotted as the mean CFU per milliliter from triplicate wells.

### Indirect immunofluorescence

Coverslips were washed once with PBS and fixed overnight with 3% PFA (Electron Microscopy Sciences) in PBS, pH 7.4, at 4°C. After fixation, PFA was removed and replaced with PBS. Then, cells were incubated with 50 mM glycin/PBS, pH 7.4 (Sigma-Aldrich), for 10 min and permeabilized with 0.05% saponin/1% BSA in PBS for 10 min. Coverslips were incubated with the mouse anti–human LAMP-2 antibody (Hybridoma Bank) at a dilution of 1:100 for 1 h at room temperature, followed by the goat anti–mouse Alexa Fluor 546 antibody (Jackson ImmunoResearch Laboratories, Inc.) at a dilution of 1:800 for 1 h at room temperature. Nuclei were stained with DAPI, and coverslips were mounted with mounting medium (Dakok). Samples were analyzed using a laser-scanning confocal microscope (SP5; Leica Biosystems).

For analysis, RGB images or frames were split into separate color channels. Bacteria (green channel) were thresholded per single cell, and the mean fluorescence intensity of LAMP-2 associated to the phagosome was measured for each frame by redirecting measurements to the channel of interest. Fluorescence intensity values were plotted using Prism software (GraphPad Software). The number of GFP-positive pixels per cell (or a value called RAWIntDen, which correlates to the number of GFP-positive pixels multiplied by 255) was plotted as a measure of the intracellular bacterial replication over time. The proportion of infected cells per sample was also determined.

### Quantification of bacterial burden with cell death inhibitors

Out-of-focus cells were manually cropped from tile scans, and the GFP per cell was quantified with an in-house R script using the RBioFormats (Oleś, 2014) and EBImage (Pau et al., 2010) packages (Fig. S3 B). For each image, nuclei were identified by DAPI staining, segmented by watershed, and used as seeds for a Voronoi segmentation of the field by Euclidian distance. Pixels in the GFP channel above a predetermined threshold were counted per segment, and segments with ≥5 GFP-positive pixels were considered infected cells. A minimum of 349 cells were analyzed per condition across three biological replicates, with a mean of 1,450.

### PM integrity in fixed cells

Before collecting the coverslips, the macrophages were stained by 25 µg/ml PI (Sigma-Aldrich) in RPMI for 15 min at room temperature and fixed in 3% PFA overnight at 4°C. Images were obtained using an SP5 confocal microscope with an argon laser emitting dually at 488 nm for excitation of GFP and at 561 nm for PI. To determine the replication of bacteria in PI-positive or -negative cells, bacteria (green channel) were thresholded per single cell, and the pixel numbers were measured in each positive and negative propidium cell. Values were plotted using Prism software.

### Live-cell imaging

For live-cell imaging, 10^5^ macrophages were seeded on 35-mm glass-bottom dishes (MatTek Corporation) and treated overnight with 100 U/ml IFN-γ in RPMI complete media. Cells were washed with PBS and infected with *M. tuberculosis* at an MOI of 1 for 2 h. After infection, cells were washed with PBS and replaced with RPMI complete media. 24 h after infection, the media were replaced with imaging medium: complete RPMI medium with 0.4 µg/ml PI. *M. tuberculosis* was also grown in imaging medium alone by simply adding the infection inoculum into a glass-bottom dish containing no macrophages. Imaging was performed with an SP5 laser-scanning confocal microscope equipped with an environment control chamber (EMBLEM). During imaging, a single focal plane was monitored in time (xyt scanning mode) using a 63×/1.4 NA HCX-PLAPO oil objective, a 488-nm argon laser, and a 561-nm diode-pumped solid-state (DPSS) laser (scanner frequency 400 Hz; line averaging 2) using photomultiplier detectors and/or hybrid detectors (HyDs) at a scanning resolution of 1,024 × 1,024 pixels. Analysis was performed using FIJI (ImageJ; National Institutes of Health). For analysis, RGB images or frames were split into separate color channels. Bacteria (green channel) were thresholded per single cell. Pixels were quantified and plotted using Prism software. Bacteria replication was calculated in PI-positive and -negative cells for each time point of interest.

### EM

GM-CSF and M-CSF macrophages were prepared as described in the Preparation of monocyte-derived macrophages section (2 × 10^6^ cells seeded per T25 flask) and infected at an MOI 1 for 5 h, with 200 ng/ml IFN-γ added after infection where appropriate. The protocol for resin embedding was adapted from Rohde et al. (2012). Samples were fixed by adding warm 2% glutaraldehyde in 200 mM Hepes, pH 7.4, directly to the cell culture medium at a 1:1 volume ratio. After 5 min, the mixture of the fixative and medium was replaced with 1% glutaraldehyde in Hepes buffer, and the samples were incubated overnight at 4°C. Cells were scraped and embedded in 1% low melting point agarose. Agarose blocks were postfixed with 2% osmium tetroxide solution containing 1.5% potassium ferricyanide for 1 h on ice and then stained with 0.5% tannin for 20 min and with 1.5% aqueous uranyl acetate for 1 h. Next, cells were dehydrated at room temperature using a graded ethanol series (70, 80, 90, 95, and 100%), followed by gradual infiltration with Spurr’s resin (Polysciences) over 2 d. Ultrathin sections (∼70 nm) were cut with an ultramicrotome Ultracut EM UCT ultramicrotome (Leica Microsystems) and contrasted with 0.2% lead citrate. Cells were examined with a transmission electron microscope (JEM-1400; JEOL). The images were recorded digitally with a TemCam-F216 camera (TVIPS). Between 24 and 89 infected cells per sample were imaged by systematic and random sampling. Cross points of the stereological test grid over bacteria were counted with regard to the subcellular localization of bacteria, and fractions of membrane-bound and cytosolic bacteria were calculated from total counts per sample. The data were plotted as the proportion of *M. tuberculosis* in the cytosol versus the proportion in membrane-bound compartments for each condition.

### CLEM

GM-CSF macrophages were prepared as described in the Preparation of monocyte-derived macrophages section, and cells were seeded to ∼30–50% confluence onto gridded dishes (MatTek Corporation) in 400 µl RPMI + 10% FCS for a 2-h attachment time at 37°C and 5% CO_2_. They were then infected with GFP-Mtb at an MOI of 1 as described in the Infection of human monocyte-derived macrophages section and incubated for 24 h. The infection medium was then replaced with 2 ml RPMI + 10% FCS containing 0.4 µg/ml PI, and then the dish was securely fastened into a custom-made dish holder for the stage of a confocal microscope ready for imaging. The microscope was set up with the argon laser set to 20% and the DPSS laser switched on. A sequential scan for each channel was set up: (a) argon 488-nm laser (set to 10%) with the HyD in brightR mode; or (b) DPSS 561-nm laser (set to 2%) with the HyD in brightR mode, and simultaneously a brightfield image was obtained using SCAN-BF mode with gain set to 493 V. Initially, a 10× objective was used to determine the grid reference of the cell of interest. A 63× oil objective was then used with the following imaging conditions: 1,024 × 1,024–pixel resolution; line averaging 2; zoom 1; and frames every 15 min. At the desired time point, warm double-strength fixative (8% PFA in 0.2 M phosphate buffer, pH 7.4) was directly added to the cell culture medium (1:1 ratio) for 15 min at room temperature. The cell of interest was relocated and imaged. The fixative was then removed, and a 1-mm layer of 4% low melting point agarose (Sigma-Aldrich) in 0.1 M phosphate buffer was added to form a protective layer over the cells. This was placed on ice quickly to solidify. Next, 4% PFA in 0.1 M phosphate buffer was added for 15 min at room temperature, and again the cells were imaged. Finally, the fixative was removed before an overnight fixation at 4°C in 2.5% glutaraldehyde and 4% PFA in 0.1 M phosphate buffer. The cells were again imaged before processing for serial block face scanning EM (SBF SEM) commenced, using a modified National Center for Microscopy and Imaging Research protocol (Lerner et al., 2016). Coverslips were postfixed in 2% osmium tetroxide/1.5% potassium ferrocyanide for 1 h on ice, incubated in 1% wt/vol thiocarbohydrazide for 20 min before a second staining with 2% osmium tetroxide, and then incubated overnight in 1% aqueous uranyl acetate at 4°C. Cells were stained with Walton’s lead aspartate for 30 min at 60°C and dehydrated through an ethanol series on ice, incubated in a 1:1 propylene oxide/Durcupan resin mixture, and then embedded in Durcupan resin according to the manufacturer’s instructions (TAAB Laboratories Equipment Ltd.). SBF SEM images were collected using a 3View 2XP system (Gatan Inc.) mounted on a Sigma VP scanning electron microscope (ZEISS). Images were collected at 1.8 kV using the high-current setting with a 20-µm aperture at 6 Pa chamber pressure and a 2-µs dwell time. The dataset was 33.59 × 33.59 × 0.75 µm in xyz, consisting of 15 serial images of 50-nm thickness and a pixel size of 4.1 × 4.1 nm. The total volume of the dataset was ∼846 µm^3^. After SBF SEM imaging, the sample was removed, and 70-nm sections were cut on a ultramicrotome (UC7; Leica Biosystems). Images were then collected in a transmission electron microscope (Tecnai G2 Spirit BioTwin; Thermo Fisher Scientific) using a charge-coupled device camera (Orius; Gatan Inc.). The SBF SEM dataset was segmented manually using Amira 6.0 (Thermo Fisher Scientific). The PM was manually selected by morphology with reference to transmission EM (TEM) images (in which membrane gap morphology was more readily apparent, because of the higher structural resolution). Only larger gaps (greater than ∼100 nm) were segmented to account for the reduced resolution in the SBF SEM images and to prevent false positive assignment of gaps.

### CLEM alignment

Alignment between light microscopy (LM), SBF SEM, and TEM was performed using the BigWarp plugin in Fiji. We first mapped the TEM image onto the final image of the SBF SEM image stack at full resolution, using landmarks in 2D. The warped and exported TEM image was then appended to the SBF SEM stack to form a hybrid EM stack. Note that the slightly different slice thickness of the SEM stack and single TEM slice can be essentially ignored because the axial diffraction limit of the LM image is much larger than the EM slice thickness. The LM image stack was then mapped onto the hybrid EM stack (containing both SBF SEM and TEM data) using landmarks in 3D at corresponding positions in the two stacks.

### Flow cytometry

Isolated monocytes and differentiated macrophages were incubated with an Fc receptor–blocking antibody (Human BD Fc Block; BD) on ice for 20 min, followed by staining with anti–CD119-PE (clone GIR-208; eBioscience), anti–CD86-BV421 (clone 2331), anti–CD206-APC (clone 19.2), anti–CD14-AF488 (clone M5E2), or anti–CD16-APC-H7 (clone 3G8; BD) for 20 min at room temperature in the dark. Cells were washed once in PBS, fixed in 1% PFA, and acquired on a flow cytometer (Fortessa; BD) or a FACS CyAnTM (Dako). Respective isotype controls were mouse IgG1 K-PE (clone P3.6.2.8.1; eBioscience), mouse IgG1, κ-BV421 (clone X40), mouse IgG1, κ-APC (clone MOPC-21), mouse IgG2a, κ-AF488 (clone G155-178), and mouse IgG1, κ-APC-H7 (clone MOPC-21; BD).

### Western blotting

Macrophages were washed once with PBS and lysed in NP-40 for 40 min on ice. Laemmli buffer was added, and samples were boiled for 20 min at 95°C. Cell lysates were electrophoresed in 10–20% Tris-glycine gel (Thermo Fisher Scientific) and blotted to polyvinylidine fluoride membranes. After a blocking step for 1 h at room temperature in PBS containing 5% milk, blots were incubated overnight at 4°C with a rabbit polyclonal anti-IDO antibody (Cell Signaling Technology) at a dilution of 1:1,000 in the blocking buffer and 1 h at room temperature with peroxidase-coupled secondary antibody (Promega).

### Growth curve of *M. tuberculosis* in vitro in the presence of necrosis inhibitors

GFP-Mtb was grown to the midexponential phase and backdiluted to OD_600_ 0.1 in 10 ml 7H9 complete medium containing either DMSO, IM-54, MCC950, or Nec-1s, as well as an uninoculated control (i.e., 7H9 complete medium alone). All cultures were in duplicate.

### Online supplemental material

Fig. S1 is a characterization of GM-CSF and M-CSF macrophages, including the expression of specific differentiation markers and the IFN-γ receptor. Also shown are Western blots of the IFN-γ–induced protein IDO. Fig. S2 (A–C) measures the GFP per cell, the proportion of infected cells, and the proportion of PI-positive cells in resting and activated GM-CSF and M-CSF macrophages after infection with *M. tuberculosis* ΔRD1. Fig. S2 D shows snapshots from Video 1 with a quantification of *M. tuberculosis* ΔRD1 replication in resting GM-CSF macrophages. Fig. S2 E shows EM images of *M. tuberculosis*–infected GM-CSF or M-CSF macrophages after 5 h plus 2 h of infection to show the detailed ultrastructure of membrane-bound versus cytosolic bacteria. Fig. S3 A measures the growth of *M. tuberculosis* in complete 7H9 medium, including 10-µM concentrations of various cell death inhibitors, to show that they are not toxic to *M. tuberculosis* in vitro. Fig. S3 B is a demonstration of the semiautomated workflow used in Fig. 5 to quantify the GFP per cell of infected macrophages. Video 1 shows the replication of GFP-Mtb ΔRD1 in resting GM-CSF macrophages. Video 2 shows the replication of GFP-Mtb in activated GM-CSF macrophages (some of which are positive for PI).

## Supplementary Material

Supplemental Materials (PDF)

Video 1

Video 2
