# Supplementary material for: Mycobacterium tuberculosis replicates within necrotic human macrophages
Source: J Cell Biol. 2017 Mar 6;216(3):583–94. doi: 10.1083/jcb.201603040 (PMC5350509; doi:10.1083/jcb.201603040)
Supplement: Supplemental Materials (PDF) [file JCB_201603040_sm.pdf]

Lerner et al., https://doi.org/10.1083/jcb.201603040

THE JOURNAL OF CELL BIOLOGY

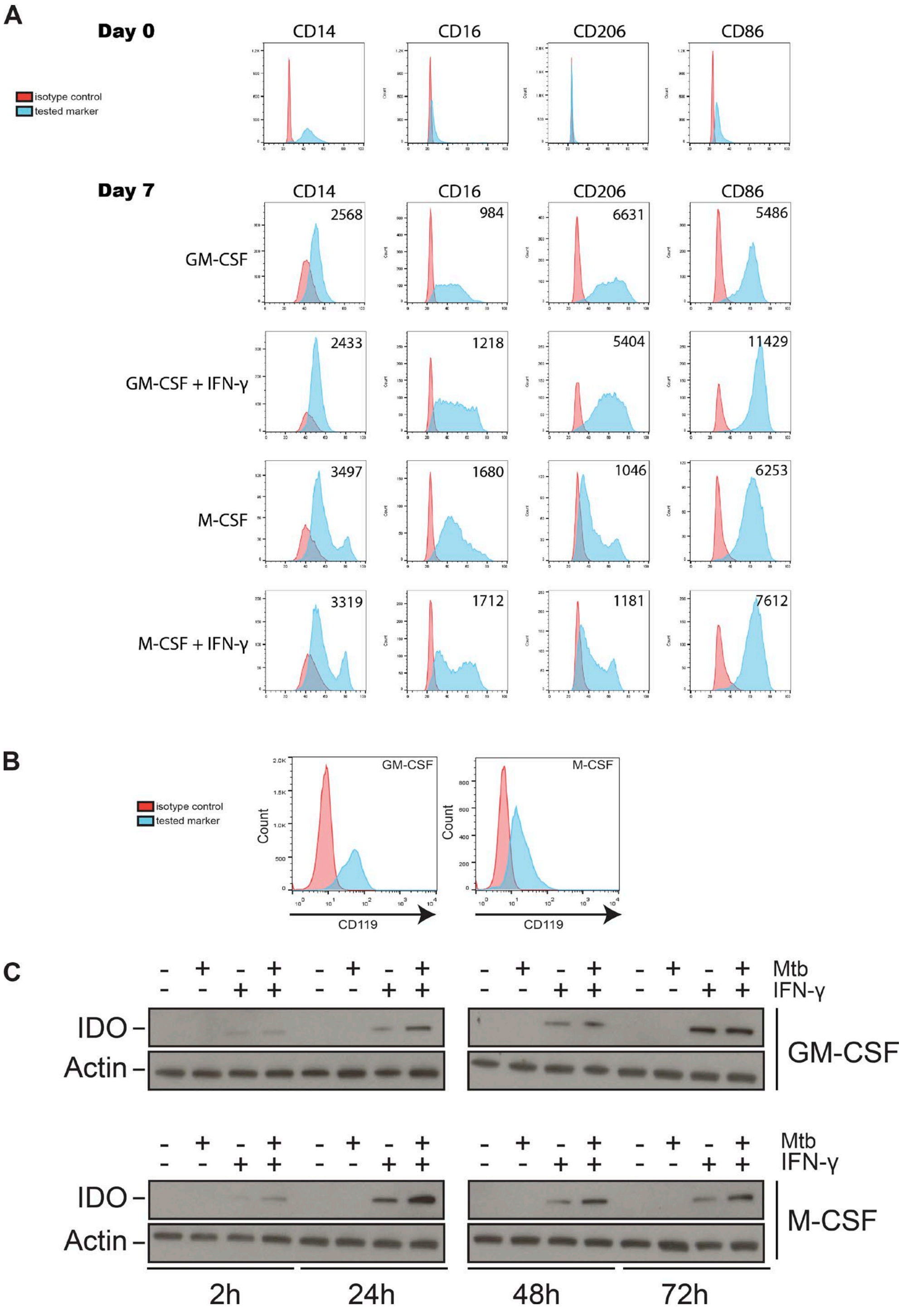


Figure S1. **Characterization of the GM-CSF and M-CSF macrophage populations.** (A) GM-CSF– and M-CSF–treated macrophages were stained with CD14, CD16, CD206, and CD86 to assess maturation and differentiation. Macrophages differentiated as expected as CD14 expression is down-regulated after treatment and CD16 expression is up-regulated. Differential responses to M-CSF and GM-CSF are highlighted by the expression of CD206, which is highly expressed on GM-CSF– but not on M-CSF–differentiated macrophages. Both macrophage types show a mature phenotype as they express CD86 to a high degree. Numbers in the top right corner of each graph indicate median fluorescence intensities. (B) GM-CSF– and M-CSF–treated macrophages were stained with anti–human CD119 antibody to assess IFN-γ receptor expression. Macrophages were preactivated with 100 U/ml IFN-γ where indicated. Both populations showed that the IFN-γ receptor is expressed. (C) GM-CSF and M-CSF cells were prestimulated or not prestimulated with IFN-γ, collected, and lysed at different time points of infection. Cells were blotted with IDO antibody or actin antibody as a loading control.

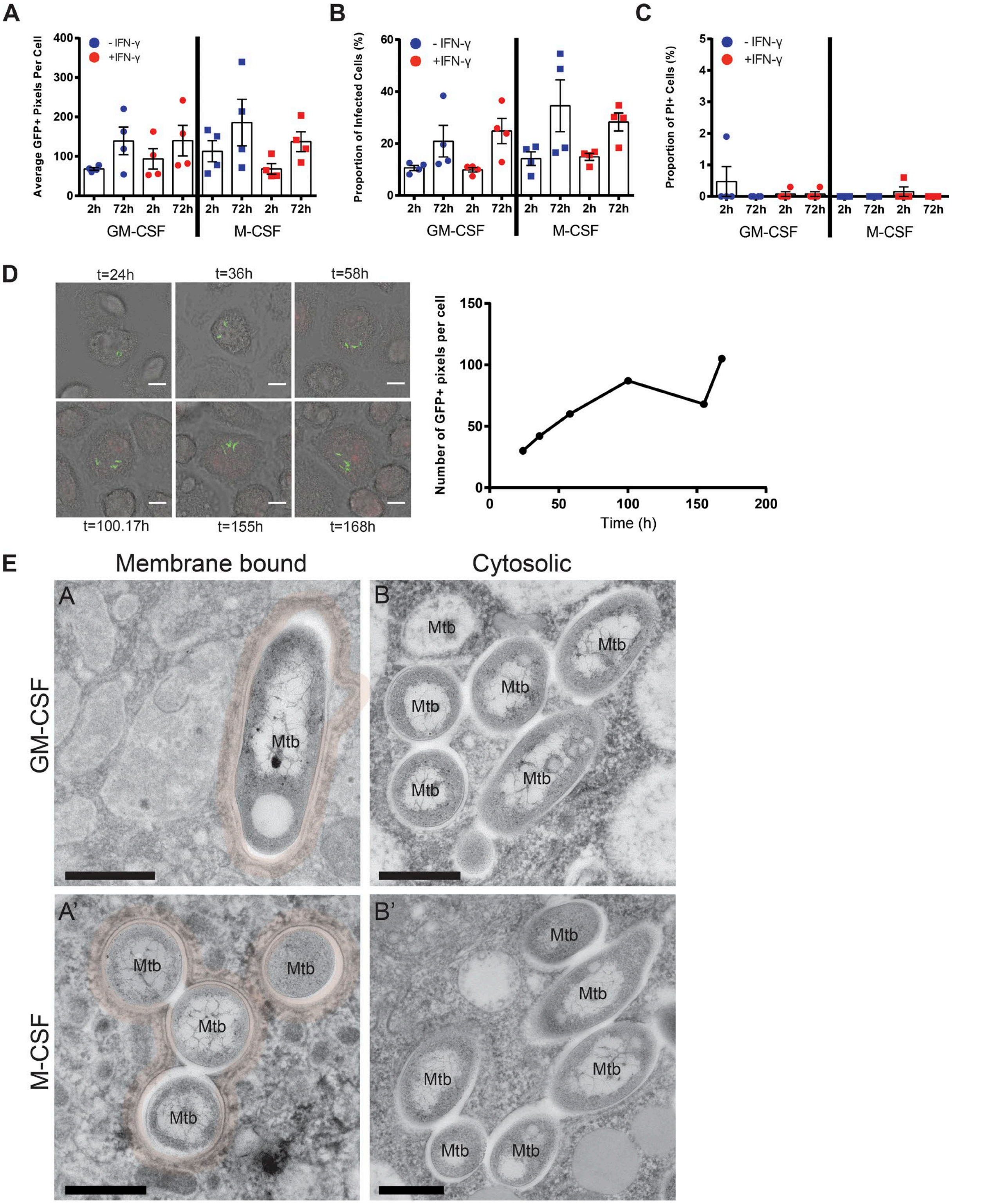


Figure S2. **Growth of *M. tuberculosis* ΔRD1 in GM-CSF and M-CSF macrophages and *M. tuberculosis* wild type is present in the cytosol even after 5 h plus 2 h of infection.** (A) Quantification of the intracellular replication of GFP-Mtb ΔRD1 from 2 to 72 h in resting (blue) or activated (red) GM-CSF (dots) or M-CSF (squares) macrophages. The mean number of GFP-positive pixels per cell was calculated for each condition, with one data point per independent experiment. (B) Quantification of the mean proportion of GFP-Mtb ΔRD1–infected cells in resting (blue) or activated (red) GM-CSF (dots) or M-CSF (squares) macrophages at 2 and 72 h after infection. (C) Quantification of the proportion of cells that had a PI-positive (PI+) nucleus in resting (blue) or activated (red) GM-CSF (dots) or M-CSF (squares) macrophages at 2 and 72 h after infection. Error bars show means ± SEM from four independent experiments. (D) Images of the first and last frame from a video in which resting GM-CSF macrophages were infected with GFP-Mtb ΔRD1 at MOI 1 for 2 h (see Video 2). PI was added to the medium after 24 h, when the live-cell imaging commenced. The graph shows the bacterial replication in one representative cell over time. (E) Example TEM images of GFP-Mtb–infected resting GM-CSF or M-CSF macrophages at 2 h after infection (excluding 5-h uptake). Surrounding host membranes are highlighted in brown, and individual *M. tuberculosis* cells are labeled. Bars, 500 nm.

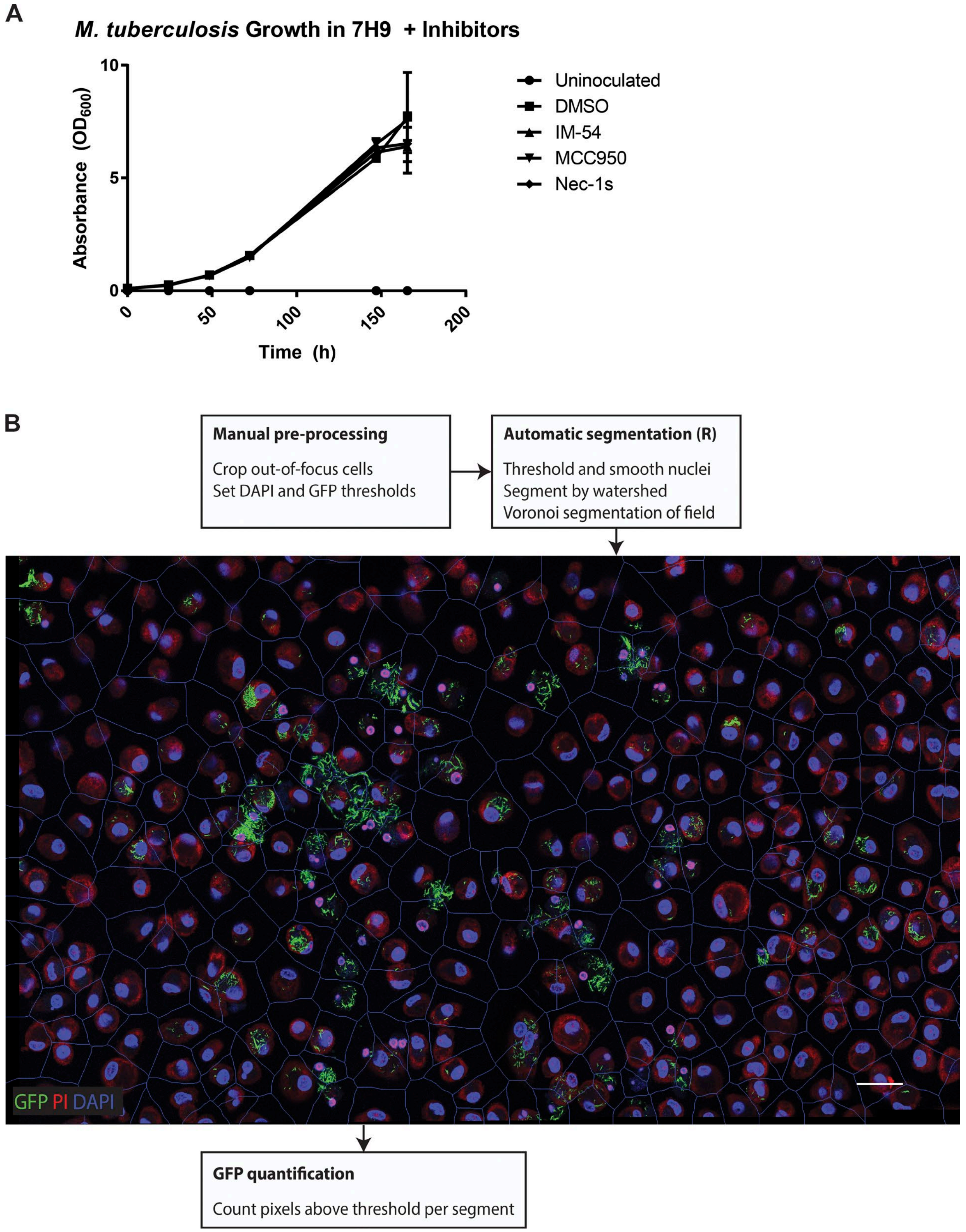


Figure S3. **Graph showing that necrosis inhibitors do not affect GFP-Mtb growth in vitro, and a schematic of automated GFP per cell quantification showing a typical field segmentation.** (A) GFP-Mtb was grown in 7H9 broth in the presence or absence of the necrosis inhibitors IM-54, MCC950, or Nec-1s (all at 10-μM final concentrations). $OD_{600}$ readings were taken over time in duplicate cultures, and means ± SEM are plotted. (B) Schematic of automated GFP per cell quantification showing a typical field segmentation. Bar, 40 μm.

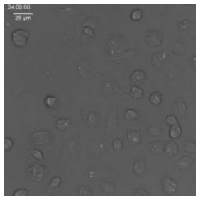

Video 1. **Replication of GFP-Mtb ΔRD1 in resting GM-CSF cells.** GM-CSF cells were infected with GFP-Mtb with an MOI of 1 for 2 h. Then, cells were washed, and after 24 h of infection, the media were replaced with complete RPMI medium with 0.4 µg/ml PI as an imaging medium. Bar, 25 µm. This video was shot at ten frames per second.

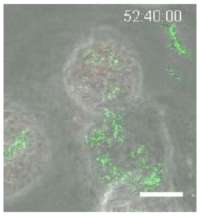


Video 2. **Replication of GFP-Mtb in GM-CSF cells treated with IFN-γ after infection.** GM-CSF cells were infected with GFP-Mtb with an MOI of 1 for 2 h. Then, cells were washed, and IFN-γ was added at a concentration of 100 U/ml. 48 h after infection, the media were replaced with complete RPMI medium with 0.4 µg/ml PI as an imaging medium. Bar, 10 µm. This video was shot at ten frames per second.
